# Ethnic differences in hepatocellular carcinoma prevalence and therapeutic outcomes

**DOI:** 10.1002/cnr2.1821

**Published:** 2023-06-21

**Authors:** Vivek Chavda, Kelsee K. Zajac, Jenna Lynn Gunn, Pankti Balar, Avinash Khadela, Dixa Vaghela, Shruti Soni, Charles R. Ashby, Amit K. Tiwari

**Affiliations:** ^1^ Department of Pharmaceutics and Pharmaceutical Technology L M College of Pharmacy Ahmedabad India; ^2^ Department of Pharmacology and Experimental Therapeutics, College of Pharmacy and Pharmaceutical Sciences University of Toledo Ohio USA; ^3^ Pharmacy Section L M College of Pharmacy Ahmedabad India; ^4^ Department of Pharmacology L M College of Pharmacy Ahmedabad India; ^5^ PharmD Section L M College of Pharmacy Ahmedabad India; ^6^ Department of Pharmaceutical Sciences, College of Pharmacy St. John's University New York New York USA; ^7^ Department of Cancer Biology, College of Medicine and Life Sciences University of Toledo Toledo Ohio USA

**Keywords:** hepatocellular carcinoma, liver cancer, racial disparity, therapeutic outcomes

## Abstract

**Background:**

Hepatocellular carcinoma (HCC) is a leading cause of cancer‐related death worldwide. The incidence of HCC is affected by genetic and non‐genetic factors. Genetically, mutations in the genes, tumor protein P53 (TP53), catenin beta 1 (CTNNB1), AT‐rich interaction domain 1A (ARIC1A), cyclin dependent kinase inhibitor 2A (CDKN2A), mannose 6‐phosphate (M6P), smooth muscle action against decapentaplegic (SMAD2), retinoblastoma gene (RB1), cyclin D, antigen presenting cells (APC), AXIN1, and E‐cadherin, have been shown to contribute to the occurrence of HCC. Non‐genetic factors, including alcohol consumption, exposure to aflatoxin, age, gender, presence of hepatitis B (HBV), hepatitis C (HCV), and non‐alcoholic fatty liver disease (NAFLD), increase the risk of HCC.

**Recent Findings:**

The severity of the disease and its occurrence vary based on geographical location. Furthermore, men and minorities have been shown to be disproportionately affected by HCC, compared with women and non‐minorities. Ethnicity has been reported to significantly affect tumorigenesis and clinical outcomes in patients diagnosed with HCC. Generally, differences in gene expression and/or the presence of comorbid medical diseases affect or influence the progression of HCC. Non‐Caucasian HCC patients are significantly more likely to have poorer survival outcomes, compared to their Caucasian counterparts. Finally, there are a number of factors that contribute to the success rate of treatments for HCC.

**Conclusion:**

Assessment and treatment of HCC must be consistent using evidence‐based guidelines and standardized outcomes, as well as international clinical practice guidelines for global consensus. Standardizing the assessment approach and method will enable comparison and improvement of liver cancer research through collaboration between researchers, healthcare providers, and advocacy groups. In this review, we will focus on discussing epidemiological factors that result in deviations and changes in treatment approaches for HCC.

## 1 INTRODUCTION

In 2020, primary liver malignancy was the third most common cause of cancer related death among men and women worldwide and 75%–85% of those deaths were attributed to HCC.^1^ Over the past few decades, the incidence rates of HCC have steadily increased in many countries, whereas the overall 5—year survival rate is less than 20%.^2^, ^3^ It has been estimated that the incidence of HCC will surpass one million people annually by the year 2025.^4^ At diagnosis, HCC is frequently associated with a poor prognosis due to detection of advanced stage tumors.^5^ This is partly due to the lack of timely diagnostic biomarkers, obvious symptomology in earlier stages, and a low frequency of radical resectable HCC at the time of diagnosis.^6^ Clinical studies have reported a significant difference in the progression of HCC based on ethnicity.^7^ Regardless of etiology, approximately 90% of HCC cases occur in the setting of chronic liver inflammation.^8^ Prior to the development of HCC, a sustained inflammatory response leads to the concurrent remodeling and regeneration of hepatocytes, which causes fibrotic deposition that eventually progresses to cirrhosis.^9^ Globally, chronic liver damage caused by infection with hepatitis B (HBV) or C virus (HCV) accounts for the majority of HCC cases.^10^ The likelihood of HCC increases with chronic alcohol ingestion, consumption of aflatoxin, nonalcoholic fatty liver disease (NAFLD), nonalcoholic steatohepatitis (NASH), hemochromatosis, and alpha‐1‐antitrypsin deficiency.^11^ Comorbid conditions, such as diabetes mellitus, obesity, and metabolic syndrome, also facilitate the progression of HCC.^12^ Notably, the prevalence of diabetes and obesity was significantly higher in patients with HCC, compared to patients diagnosed with viral and alcohol cirrhosis.^13^

**FIGURE 1:**
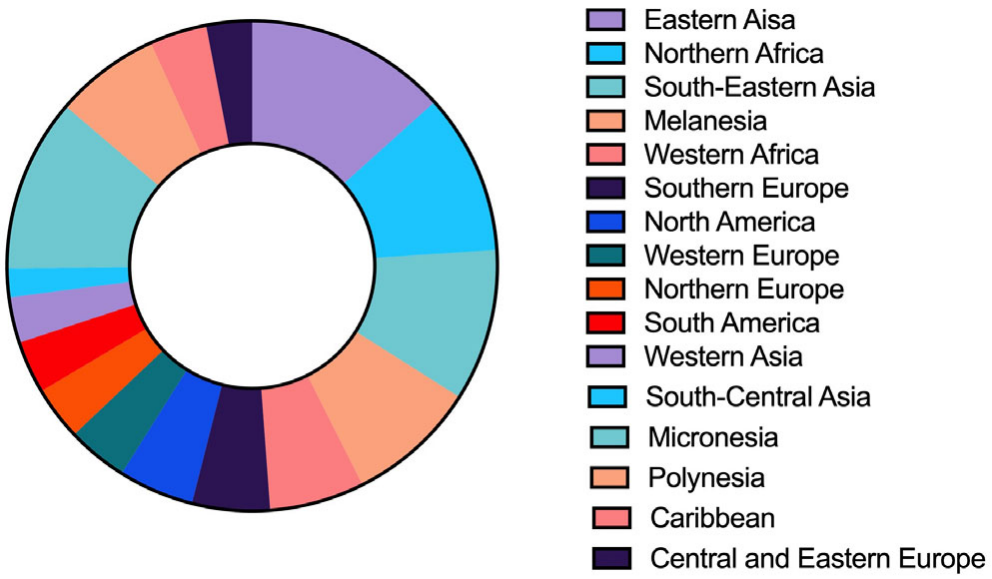
Geographical prevalence of HCC. The prevalence of HCC varies geographically, which may be due to differences in predisposing factors that increase the risk of HCC.^14^, ^15^

Differences in ethnicity and geography have been shown to significantly affect all‐cause mortality and increase the risk of HCC.^16^ The majority of HCC cases and the lowest survival rates after treatment primarily occur in Asia and Sub‐Saharan Africa, predominately due to HBV infection, whereas HCV infections are the most common cause in the United States.^17^ Figure 1 illustrates the global prevalence of HCC. HCC disproportionately affects disadvantaged minorities, leading to disparities in the initial detection, stage at diagnosis, and overall mortality rate.^18^ A National Health and Nutrition Examination Survey reported that in the United States, individuals of African‐American descent living in southern states are more likely than other ethnicities to be diagnosed with chronic HCC.^19^ Asians have the highest overall incidence of HCC, followed by African‐Americans, Hispanics, and non‐Hispanic Caucasians.^20^ HBV may be further classified into its 10 known genomes (A–J) and emerging research indicates that certain HBV genotypes play a role in determining clinical outcomes.^21^, ^22^, ^23^ Native Alaskans have higher rates of HCC when infected with HBV genotypes A, C and F, compared to patients with genotype B or D.^24^ Disparities in the incidence of HCC are also significantly correlated with an individual's age, sex, socioeconomic status, and place of residence.^25^ Individuals of African descent have a significantly higher mortality rate from HCC compared to other ethnic groups.^26^ Mathur et al.^12^ reported that the mortality rate in these individuals are 12%, 10%–12% and 16% greater than Caucasians, Hispanics, and Asians, respectively.^27^ There are significant differences in disease burden, treatment, and outcomes of HCC, based on ethnicity and gender.^28^ Consequently, early detection, high‐quality prevention, and evidence‐based treatment will be necessary to significantly decrease these HCC disparities.^29^

This review aims to discuss the epidemiology of ethnic and geographical disparities in patients who have been diagnosed with HCC, the gene–environment parameters, along with the types of treatments that have been shown to affect survival rates among these patients.^30^

## 2 EPIDEMIOLOGY OF HCC

**FIGURE 2:**
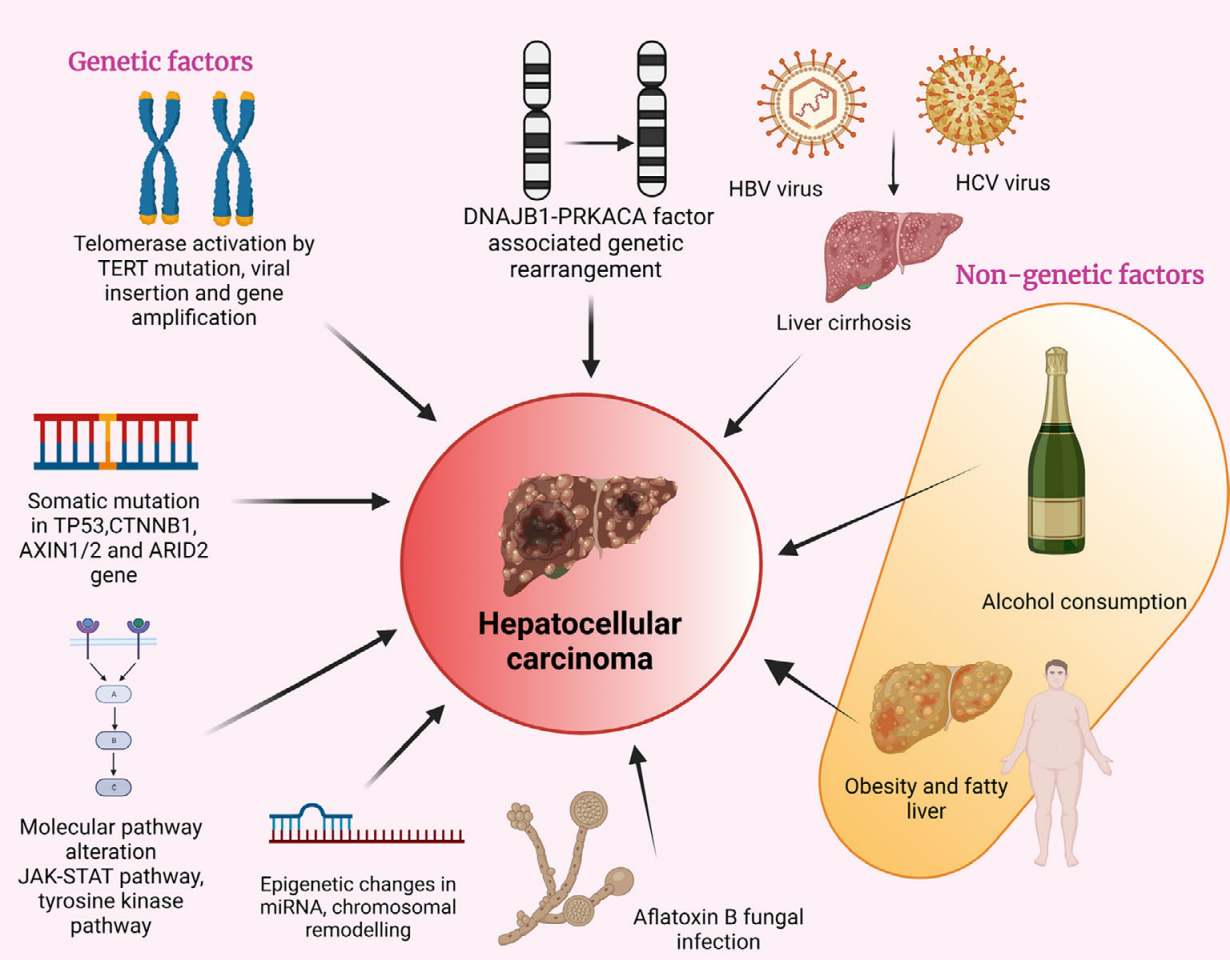
Factors affecting the risk of developing HCC. Factors influencing the development of HCC can primarily be classified as genetic or nongenetic factors. Genetic factors include telomerase reverse transcriptase (TERT) mutations, somatic mutations, alterations in the Janus kinase (JAK)‐Signal transduction and activation of transcription (STAT) pathway and chromosomal remodeling. The nongenetic factors include HBV, HCV, alcohol consumption, obesity, fatty liver disease, exposure to aflatoxin and liver cirrhosis, among others.

The majority of hepatocellular carcinoma (HCC) cases are due to chronic HBV and HCV infection.^31^ Host variations in age, genetics, gender, as well as environmental factors, affect the ethnic‐specific HCC rates in different geographical regions. These are likely due to differences in the severity and infectious capacity of HBV and HCV.^15^ (Figure 2).

### 2.1 Hepatitis B virus (HBV)

HBV is a DNA virus transmitted by exposure to infectious blood or bodily fluids which includes vertical transmission. Globally, HBV is the leading causative factor of HCC.^32^ Yearly, there are approximately 60 000 new cases of HBV in the United States, with approximately 75% of the 350–400 million worldwide population with chronic HBV being of Asian descent.^33^, ^34^, ^35^ A productive HBV infection leads to HCC development through direct and indirect mechanisms. HBV infection can  induce inflammation which causes continuous necrosis of infected hepatocytes or uncontrollably stimulate the proliferation of adult hepatocytes.^37^ HBV primarily affects the functionality of healthy hepatocytes by promoting replication which leads to liver damage, fibrosis, and cirrhosis in conjunction with the host immune response. A whole genome sequencing study in HBV‐associated HCC patients was conducted to identify any genetically altered pathways that could be correlated with HCC progression.^36^ There was a significant correlation between the expression of SYT12, GATM and FN1 in patients with early onset HBV‐related HCC, whereas there was a significant correlation in the expression of STAT1, ALB, MLL4 and TERT in patients with late onset HCC.^39^ Additionally, HBV induces HCC formation through the activation or deactivation of various tumorigenic pathways. In the normal liver, β‐catenin is a membrane localized protein which can be activated via HBV on hepatocytes. There is potential that activation of this pathway may be involved in the somatic genetic and nongenomic events in HCC.^37^ Activation of β‐catenin signaling is considered an early event in HCC pathogenesis. Prominent signaling can lead to the overexpression of β‐catenin which results in the proliferation of cancer stem cells in HCC.^38^ Mutations within the gene coding for ß‐catenin (CTNNB1) was observed in 20%–35% of primary HCC patients.^39^ HBV can damage DNA and create mutations within cancer promoting genes such as TP53. Oncogenic activation of certain genes can ultimately lead to tumor progression.^40^ One study's results indicated that among 81 patients, the most frequently mutated oncogene was beta‐catenin (15.9%) and the most frequently mutated tumor suppressor gene was TP53 (35.2%). The two major oncogenic mutations linked to the progression of HCC were the Wingless‐related integration site (Wnt)/beta‐catenin and JAK/STAT pathways, which were altered in 62.5% and 45.5% of patients, respectively.^36^ The risk of HCC development is significantly increased in patients with higher rates of HBV replication.^41^ The incidence of HCC and mortality rates significantly increase when a country's prevalence of HBV is greater than 2%.^42^ The annual risk of HCC is 0.5% for patients that are asymptomatic but express the protein hepatitis B serum antigen (HBsAg) and 0.8% for patients with chronic HBV.^43^ Chronic HBV carriers have a 10%–25% risk of developing HCC with 80%–90% of HBV‐related HCC cases occurring in patients with cirrhosis.^31^, ^42^ Notably, HBV‐induced cirrhosis produces a 100‐fold greater risk of developing HCC, compared to HBV infected patients without cirrhosis.^4^

### 2.2 Hepatitis C virus (HCV)

HCV infection is another risk factor that increases the likelihood of HCC. The estimated global prevalence of HCV is approximately 2.5%, with the highest prevalence in Central Asia and Central Africa.^44^ Globally, it has been estimated that approximately 170 million people are infected with HCV, and this varies among geographical regions.^45^ From 1990 to 2005, there was a decrease in the incidence of HCV infected patients in certain higher‐income areas, such as Western Europe, Southern Africa, and Australia; however, there was a significant increase in the incidence of HCC in lower‐income areas, such as Central Africa and Central Asia.^44^ Egypt has one of the highest rates of HCC in the world, which was primarily due to the parenteral treatment of schistosomiasis from 1950 to 1980. The reuse of needles that were inadequately sterilized during this time increased the transmission of blood‐borne pathogens, including HCV.^46^ Long‐standing HCV may progress to liver fibrosis and cirrhosis, which increases the risk of HCC.^47^ Approximately 15% – 40% of patients spontaneously clear HCV, with 50% – 80% developing a chronic infection, 20% – 30% developing cirrhosis or liver failure, and 2% – 5% developing HCV‐induced HCC.^45^, ^47^, ^48^ HCV is a major etiological factor for HCC, and studies indicated that at least 70% of patients with HCC have serum anti‐HCV antibodies.^49^ Patients that are infected with HBV and HCV have a significantly higher risk of developing HCC compared to than those infected with HBV or HCV alone.^50^, ^51^ The proteins that have oncogenic potential in HCC are the core, nonstructural 3 (NS3), nonstructural 5A (NS5A) and nonstructural 5B (NS5B).^52^ Clinical studies have shown that the levels of these proteins are positively correlated with an increased risk of HCC.^53^ It has been hypothesized that HCV increases the risk of HCC by producing mutations that create chronic inflammation, by increasing the levels of certain pro‐inflammatory cytokines, cell‐cycle dysregulation, and inhibition of the transcription of tumor‐suppressor genes.^54^ HCV infection causes hepatocarcinogenesis by inducing the progressive accumulation of genetic mutations, which promotes malignant transformation, and by increasing hepatocyte turnover through chronic liver damage, which creates inflammatory and oxidative stress.^55^ HCV infection induces inflammation within the liver, causing the accumulation of lymphocytes, an increase in the levels of reactive oxygen species (ROS), and certain cytokines.^56^ The chronic inflammation produced by HCV can produce fibrinogenesis and hepatic stellate cell activation, angiogenesis, DNA damage and mutations due to dysfunctional apoptosis, thereby increasing the risk of HCC.^54^, ^57^

### 2.3 Aflatoxin B_1_

Aflatoxins are naturally occurring carcinogens produced by *Aspergillus flavus* and *parasiticus* that can contaminate food supplies of human and animals such as maize, wheat, and peanuts.^58^ The consumption of aflatoxins can cause hepatotoxicity and immunotoxicity^59^ and the most potent of these compounds is aflatoxin B_1_ (AFB1).^60^ Aflatoxin is recognized as a “Group A” because it can produce a significantly increase in the occurence of HCC. Aflatoxin consumption primarily occurs in countries that have warm and humid environments and thus exposure to AFB1 is most common in Southeast Asia, South America, and Sub‐Saharan Africa.^61^ A meta‐analysis estimated that AFB1 alone increases the risk of HCC by 6‐fold and exposure to AFB1 and HBV synergistically increases the risk of HCC by 54‐fold.^62^ In China, the replacement of maize with rice as a dietary staple decreased the incidence of HCC.^63^ One study suggested that mutations of p53 contribute to the development of HCC approximately half of the time. AFB1 produces the mutation of G to T at codon 249 of the p53 gene, which leads to serine being replaced by arginine.^64^ This mutation ultimately decreases the anti‐tumor efficacy of the p53 gene, which increases the development of HCC.^65^

### 2.4 Genetic susceptibility

Ethnic and geographical disparities in the incidence of HCC are correlated with genetic factors that produce differences in the severity of HCC.^28^ These genes can be separated into four different categories, depending on their function within the cell. The genes implicated in the pathogenesis of HCC include the regulation or modulation of: (1) DNA damage response (p53); (2) growth inhibition and apoptosis mannose 6‐phosphate (M6P), Mother against decapentalpegic homolog 2 (SMAD2); (3) cell cycle control (Retinoblastoma transcriptional corepressor 1 (RB)1, cyclinD) and (4) cell–cell interactions (Adenomatous polyposis coli (APC; also known as deleted in polyposis 2.5) and E‐cadherin).^66^ Alterations in these genes are typically due to single nucleotide and copy number polymorphisms, which increase the susceptibility of developing and progressing to HCC.^67^ Aberrant activation of telomerase by mutations secondary to chromosome translocations or gene amplification, are the most common genetic alterations that cause HCC.^68^ Hereditary diseases, such as hemochromatosis (HFE), tyrosinemia (FAH), alpha‐1‐antitrypsin deficiency, porphyrias (HMBS, UROD), glycogen storage diseases, and Wilson's disease increase the probability of developing HCC.^69^ Furthermore, mutations in the p36.22 region of chromosome 1, which contains genes that code for certain tumor suppression proteins, can increase the risk of HCC (Table 1). Patients with HCC have numerous gene mutations. TP53 and CTNNB1 are the most commonly mutated genes (by 29.1% and 28.6% of total cases, respectively).^70^ Aflatoxin and vinyl chloride cause epoxidation, which is responsible for the TP53 mutation. By introducing vinyl chloride, A: T is inverted to T: A.^71^ Subsequent responsible mutation present in HCC were AX1N1 (7.5%), ARID2 (8.2%), ALB (10.2%), APOB (9.8%), and ARID1A (8.8%). The ARID2 and ARID1A leads to remodeling of regulatory mechanisms which also occur in other cancers such as ovarian, lung and pancreatic cancer.^72^ The mutation predisposes to chronic liver disease primarily due to iron overload, copper overload, and metabolic disturbances. A mutation in HNF1A may develop into benign tumors with the potential to convert to malignancies.^73^

**TABLE 1:** Genetic mutation associated with HCC.

Gene mutation	Remarks	Reference
TP53 (tumor protein 53) or p53	Mutation increases the number of chromosomal rearrangements.	74
IRF2 (interferon regulatory factor 2)	The inactivation, deletion, splicing, or missense mutations within this gene increase the risk of HCC. IRF2 mutations produces hyperploidy and increases chromosomal instability. There is an increase in chromosomal proliferation, but over expression results in apoptotic cell death.	75
ARID2 (AT‐rich interactive domain)	ARID2 is a component of the PBAF complex. Mutations within this gene have been correlated an increase in the risk of HCC.	76
PNPLA3 (patting‐like phospholipase domain containing protein 3)	Mutations that decrease the activity of this enzyme causes macrovesicular steatosis and VLDL retention which increases the risk of developing HCC.	77
NFE2L2 (Nuclear factor erythroid 2‐related factor 2)	NFE2L2 codes for the protein NRF2, a transcriptional factor required to maintain the redox balance within the cell. This gene is mutated in 6.4% of HCC cases.	78, 79
KEAP1 (Kelch‐like ECH associated protein 1)	Certain mutations in NFE2L2 inhibit KEAP1—mediated degradation of ubiquitin‐proteasome, which impairs the oxidative stress response increasing the risk of HCC.	80, 81
PIK3CA (phosphatidylinositol‐4,5‐biophosphate 3‐kinase catalytic subunit alpha) and TP53	Mutations in both genes occur primarily in patients infected with HBV, which produces an increase in the viral load of HBV and severity of HCC.	82, 83
CDH8 (chromodomain helicase DNA binding protein 8) and PROKR2 (prokineticin receptor 2)	The CDH8 and PROKR2 genes mutated in 2.4% and 1.6% of HCC patients, respectively.	84
RPS6KA3 (Ribosomal protein S6 kinase A3)	RPS6KA3 gene encodes for ribosome S6 protein kinase2 (RSK2). RSK2 protein activates the RAS/MAPK pathway. Mutations in this gene inactivate RSK2 function which directly increases the risk of HCC without cirrhosis.	68, 85

### 2.5 Nongenetic factors that increase the risk of HCC

#### 2.5.1 Alcohol abuse

The chronic consumption of alcohol is a well‐established, nongenetic factor that is positively correlated with the development of HCC.^86^ Globally, alcohol‐induced liver damage is the most prevalent cause of chronic liver damage.^87^ The metabolism of ethanol creates pathologic oxidative, metabolic, and inflammatory stress that causes hepatocyte injury and dysfunction.^88^ Over time, chronic alcohol consumption may progress to steatosis, steatohepatitis, cirrhosis, and potentially HCC.^89^ In recent years, the annual consumption of alcohol per capita has increased in the United States and it has been estimated that approximately 7% of adults meet the criteria for alcohol dependence or alcohol use disorder.^90^ Furthermore, the consumption of alcohol has significantly increased in men and younger individuals in Asia.^91^ Chronic liver disease, precipitated by long‐term alcohol consumption, is responsible for 30% of HCC cases worldwide, and this number is expected to increase in the future.^87^, ^92^ Geographically, the rates of alcohol consumption vary by region, and as a result, countries with notable consumption have a higher incidence of alcohol‐associated HCC.^90^, ^93^ Recent clinical data indicate that excessive alcohol consumption in European countries accounts for 40%–50% of all liver malignancies.^94^ The Global Burden of Disease study of 2019 estimated that alcohol‐associated HCC accounted for 35%, 33%, and 27% of overall HCC mortality in Europe, North and South America, and Southeast Asia, respectively. Eastern Mediterranean and Western Pacific countries reported the lowest rates of alcohol‐associated HCC deaths at 10% and 11%, respectively.^93^ The consumption of 80 g/day for more than a decade increases the probability of HCC by 5‐fold.^57^ A study in the U.S. reported that heavy drinkers (>7 drinks per day) had an 87% increased risk of developing HCC, compared to nondrinkers.^95^ Similarly, a meta‐analysis reported a 16% increase in the likelihood of the development of HCC in individuals that consumed more than three drinks a day.^96^ In addition, there was a synergistic increase in the risk of developing HCC in people that consumed alcohol and were concurrently infected with HBV or HCV, compared to either factor alone.^97^

#### 2.5.2 Nonalcoholic fatty liver disease (NAFLD)

Clinical studies have shown that patients diagnosed with diabetes, obesity, or metabolic syndrome, who drink little or no alcohol, are significantly more likely to be develop nonalcoholic fatty liver disease (NAFLD),^98^ which can increase the risk of HCC.^99^ The pathophysiology of NAFLD results from the accumulation of fat within the liver in the absence of a secondary cause which may ultimately progress into cirrhosis.^100^, ^101^ The estimated worldwide prevalence of NAFLD is approximately 25% and the incidence continues to increase. NAFLD's prevalence parallels the obesity epidemic that is primarily observed in western countries and it is the most rapid growing indication for liver transplantation.^102^, ^103^ The rapid rise in NAFLD is projected to become the primary etiological factor for producing HCC in certain countries. One study from the United Kingdom reported a < 10% to 34.8% increase in NAFLD‐associated HCC from 2000 to 2010.^104^ Similarly, another study in France indicated that the prevalence of NAFLD‐associated HCC increased from 2.6% (1995–1999) to 19.5% (2010–2014).^105^ The prevalence of NAFLD‐associated HCC will increase by 146% from 2015 to 2030, based on a Markov model that uses historical and projected rates of diabetes and obesity in the United States.^106^ The BRIDGE international study (*n* = 18 031 from 2005 to 2011) reported that the geographical regions with the highest prevalence of NAFLD‐associated HCC were North America (12%), Europe (10%), South Korea (6%), and Taiwan (5%). Furthermore, this large international study revealed that lower rates of NAFLD‐associated HCC occurred in China (1%) and Japan (2%).^107^ Based on the predictions from Este et al.,^94^ China and Japan are projected to have an increase in NAFLD‐associated HCC by 86% and 47%, respectively.^106^

#### 2.5.3 Nonalcoholic steatohepatitis (NASH)

As NAFLD progressively worsens, it can potentially transform into its inflammatory subtype known as NASH.^108^ The transition from NAFLD to NASH is hypothesized to result from: (1) the accumulation of lipids within the liver, which increases insulin resistance and (2) chronic insulin resistance induces molecular and cellular changes within the hepatocytes, while also producing hepatic inflammation.^108^ Insulin resistance produced by NAFLD causes a compensatory hyperinsulinemic response^109^ which facilitates the development of primary liver malignancy.^110^ It has been estimated that approximately 20% of all cases of NASH slowly progress to advanced cirrhosis.^111^ Risk factors associated with the development of NASH are similar to those for NAFLD, such as metabolic syndrome, obesity, and insulin resistance.^112^ Similar to NAFLD, the incidence of NASH‐associated HCC parallels the obesity epidemic and is increasing worldwide.^113^ As countries adapt a more sedentary lifestyle with poorer diets, the risk of developing NASH increases. One study in the United States reported that the prevalence of NASH increased from 1.51% in 2010 to 2.79% in 2020.^114^ The increase incidence of NASH among individuals is also expected to occur in China, India, Indonesia, Pakistan, Bangladesh and the Philippines, where the prevalence of type 2 diabetes has been increasing over the past decade.^115^ Annually, approximately 0.7% to 2.6% of patients diagnosed with NASH will develop HCC in the United States and Europe.^112^, ^116^ In a large survey of cirrhotic Japanese patients, 31.5% of patients diagnosed with NASH‐cirrhosis had HCC.^117^ HCC cases with no secondary cause are known as cryptogenic HCC. It is likely that NASH‐associated HCC is significantly underestimated, and consequently, its true prevalence remains to be determined. The identification of these cases will contribute to the increase in rise of NASH‐associated HCC.^118^, ^119^

#### 2.5.4 Obesity

Obesity has been identified as an independent risk factor for the occurrence of primary liver malignancy.^120^ Clinical data indicates that obese patients, compared to nonobese patients, have higher plasma levels of leptin, which is a proinflammatory, proangiogenic, and profibrogenic cytokine.^121^ Leptin activates the JAK pathway, which increases the probability of increase cell growth, thereby leading to cancer progression.^122^ Excess body fat has been shown to decrease the ultrasound detection of HCC and this is 3‐ to 8‐fold more likely to occur in obese patients, compared to patients of healthy weight.^123^ Finally, obese patients are significantly more likely to die from liver cancer, compared to individuals of normal weight.^124^, ^125^ As previously indicated in the NAFLD and NASH sections of this article, obesity has become an increasing problem worldwide. Obesity leads to the development of several other comorbid conditions, such as metabolic syndrome, type 2 diabetes, NAFLD and NASH, all of which have been implicated in the progression of HCC.^126^ One study from South Korea followed 700 000 men over 10 years and the results indicated that men with BMIs greater than 30, had an increased risk of developing HCC.^127^ Similarly, a North American prospective study reported that men with BMIs greater than or equal to 35, had a 4.5‐fold increased relative risk of dying from liver cancer, compared to those with normal body‐weight.^125^

#### 2.5.5 Sex disparities

It is well established that there are significant sex disparities in the incidence of HCC. Males are 2–8 time more likely to develop HCC compared to females, regardless of the geographic region and ethnicity.^128^ The prevalence of risk factors for the development of HCC in the general population has fluctuated over time, whereas the sex disparity has remained constant, suggesting that sex hormones play a role in the pathogenesis of HCC.^129^ Studies have shown that estrogen may decrease the risk of HCC by decreasing the levels of pro‐inflammatory mediators, such as IL‐6, androgens and vascular endothelial growth factor, which may decrease the progression to HCC.^130^ Men chronically infected with HBV that have hypertension and diabetes, are at a higher risk of developing HCC when their serum testosterone levels are higher, compared to individuals who are healthy.^131^ However, it has been reported that increased plasma levels of androstenedione, an androgenic compound, are correlated with a lower risk of HCC.^132^

## 3 GEOGRAPHICAL AND ETHNIC DISPARITY IN HCC

Globally, according to estimates by the World Health Organization (WHO), there were 905 677 new cases of HCC and 830 180 deaths in 2020.^133^ HCC is one of the top five most common causes of cancer‐related death in Northern and Western Africa, Eastern and South‐Eastern Asia, Central America, Western Asia, and certain countries in Europe (i.e., Bosnia, France, Romania and Italy).^133^ A study by Rumgay et al.^63^ predicted an increase in newly diagnosed cases of HCC per year by 55%, between 2020 and 2040, with 1.3 million deaths in 2040, which is 56.4% higher than the mortality rate reported in 2020.^134^ There is a significant variation in the disease burden of HCC between different ethnicities and geographical regions that parallels the geographical distribution of HBV and HCV.^135^ More than 80% of HCC cases occur in low‐income countries, notably in East Asia and sub‐Saharan Africa, where the transmission of viral hepatitis is high due to a low socio‐economic status (SES).^136^ More than half of the HCC cases in the United States of America (USA), Egypt and Pakistan, can be attributed to chronic HCV, which is the cause of 20% of all HCC cases worldwide.^137^ Furthermore, chronic HBV infection is the most prevalent cause of HCC in Central Asia, sub‐Saharan African and South‐East Asian countries.^138^

The highest incidence (54.3%) and mortality (54.1%) from HCC occurs in Eastern Asia, with Mongolia reporting 85.6 new cases per 100 000 people.^1^, ^134^ HCC is the most frequently diagnosed cancer in Vietnam, Cambodia, Thailand, Egypt, Mongolia and Laos.^139^ A recent decrease in newly diagnosed cases of HCC was reported in South Korea, China and the Philippines, due to mass immunization for HBV.^140^ However, an increase in the prevalence of risk factors, such as NAFLD obesity, diabetes, high alcohol consumption and smoking, has contributed to an increase in the incidence of HCC cases in the USA and other industrialized nations.^141^, ^142^ Furthermore, patients born between 1945 and 1965 have a higher incidence of HCC, due to a higher prevalence of HCV infection.^143^ It has been hypothesized that HCC will be the third leading cause of cancer‐related deaths in the United States, with an estimated 122% increase in incidence by 2030.^144^

There is a significant variation among countries in the age of onset of HCC.^145^ A diagnosis of HCC at an early age (30–60 years) is more common in Asiatic and the majority of African countries.^107^ In contrast, in North America, Japan, USA European nations, the median age at onset is >60 years.^139^ A study of 1552 patients with HCC from even countries in Africa reported a median age of HCC onset of 45 years.^146^ Individuals born in West Africa had the earliest age of HCC onset (>40 years).^147^ Greater than 60% of HCC cases in China, Europe, North America and Korea, are diagnosed at intermediate or advanced stages, resulting in poor outcomes.^107^ Clinical data from Japan and Taiwan indicate the optimal clinical outcomes due to regular and intensive surveillance programs for HCC.^148^ Furthermore, patients in these countries had a significantly higher median survival, compared to individuals living in sub‐Saharan Africa, where the median survival of patients diagnosed with HCC was 2.5 months.^148^, ^149^ However, the survival of patients diagnosed with HCC remains poor, even in high‐income countries.^150^ A recent study of seven high‐income countries by Rutherford et al.^78^ reported that Australia had the highest 3‐year survival rate from HCC (28%), and Denmark had the lowest (17%), between 2012 and 2014.^148^ Another study evaluating the 5‐year survival from 2010 to 2014 reported that in European countries, the survival rate was less than 10%, compared to 30% for Japan.^151^

Several studies have shown that there are significant ethnic disparities in the incidence of HCC. Mahimpundu et al.^71^ reported a higher incidence of HCC among African Americans (10.2 per 100 000), compared to Caucasian Americans (6.3 per 100 000).^143^ The etiological factors of HCC were also found to be disproportionate, as the causative factors for HCC in African Americans were tobacco and alcohol use, compared to HCV infection and diabetes for Caucasian Americans.^26^ Individuals of Hispanic ethnicity in the United States had the highest prevalence of NAFLD, with a high risk of progression to NASH.^152^ Immigration has been postulated to play a major role in the higher rates of HCC within ethnic minorities in the United States, Western Europe, Australia, and Canada.^153^

In the USA, over the past few years, there has been an increase in the incidence of HCC in Hispanic men (4.7% per year since 2000), compared to non‐Hispanic Caucasian and African‐American populations.^154^ In contrast, there has been a decrease in the incidence of HCC in middle‐aged African‐Americans (17.2% decrease per year since 2012), compared to other ethnic groups.^155^ Ethnic minorities that have a low SES and are at a higher risk of developing HCC, are not screened on a regular basis, and this increases the probability of being diagnosed at an advanced stage, resulting in a worse prognosis.^84^ Ha et al.^39^ reported that in the USA, African‐American HCC patients had higher odds [Odds ratio (OR): 1.20], whereas Asians Americans had lower odds (OR: 0.87), of having advanced stage tumors at the time of diagnosis, compared to Caucasian Americans.

The 5‐year survival rate in African Americans (21%) is significantly lower, compared to Caucasian Americans (25%), even after combining all of the stages of HCC.^143^ Data from a 2016 study in the USA indicated a descending decrease in mortality from HCC for Hispanic Americans, non‐Hispanic Asians/Pacific Islanders, Native Americans/Native Alaskans, African Americans and Caucasian Americans.^156^ In ethnic groups with a low SES, non‐Hispanic Asians/Pacific Islanders were significantly more likely to be diagnosed with HCC at a local stage, whereas Native Americans/Native Alaskans had the lowest likelihood.^25^ A significant variation in therapeutic outcomes has been reported in the USA between patients of different ethnicities.^28^ Ethnic minorities and patients with a low SES received the lowest rates of curative treatments.^157^ This occurred even in patients undergoing potentially curative treatments, such as liver transplantation and surgical resection.^157^ Furthermore, African–American patients had higher mortality rates, compared to Caucasian Americans, regardless of the SES.^158^ Data from the Surveillance Epidemiology & End Results (SEER) database indicated that African American patients had higher mortality rates (HR: 1.11), whereas Hispanic Americans had similar mortality rates (HR: 0.97) and Asian Americans had a 16% lower mortality rate, compared to Caucasian Americans.^27^ African Americans had lower odds of receiving liver transplantation, compared to patients of other ethnicities.^16^ Moreover, African–Americans had a lower 2‐ and 5‐year graft survival and higher in‐hospital mortality after liver transplantation, compared to Caucasian Americans.^28^ However, USA cancer register data suggested that the highest mortality rates occur in New York, California and Florida among Korean, Vietnamese and Chinese patients.^153^

The ethnic disparity in the outcomes of HCC, as a result of inadequate healthcare access in vulnerable populations, suggest that implementation of large‐scale interventions will be required to mitigate the mortality due to HCC.

### 3.1 Difference in epigenetics

Epigenetics involves alterations in gene expression by the external environment that occurs in the absence of changes in the DNA sequences.^159^ Alterations in epigenetic factors have been reported to be significantly correlated with the progression and outcomes in patients diagnosed with HCC.^160^ DNA methylation, histone modifications, chromatin remodeling, and noncoding RNAs, are the primary epigenetic factors affecting the carcinogenic processes that cause HCC.^161^ Furthermore, DNA hypermethylation and hypomethylation have been shown to play a role in the development of HCC. The activation of certain proto‐oncogenes and the presence of a higher number of somatic mutations, were shown to be correlated with hypomethylation.^162^ In contrast, DNA repair, angiogenesis, alterations in the metabolism of carcinogens and cell cycle regulation are significantly affected by abnormal hypermethylation.^163^ Hypermethylation leads to modifications in the WNT/ß‐catenin signaling pathway, which causes the accumulation of ß‐catenin, which increases the probability of oncogenesis.^160^, ^161^ For example, mutations in micro‐RNA (miR)‐1273f upregulates cell proliferation, migration and invasion which facilitates the prevalence of extracellular vesicles HCC.^164^ Some studies have reported a significant correlation between miRNAs and DNA methylation.^165^ miRNAs are noncoding RNA molecules composed of an average of 22 nucleotides.^166^ The combination of miRNAs and DNA methylation inactivate various tumor suppressor genes.^166^ A recent study by Varghese et al.^146^ reported ethnic disparities in the miRNA sequences and DNA methylation patterns of in liver tissue samples from individuals with HCC. The expression of miRNAs, such as miR‐150, miR‐155 and miR‐146a, were upregulated in HCV‐infected African–American patients, compared to European Americans. Has‐miR‐139‐5p, a tumor suppressor gene, was observed to be significantly downregulated in African–Americans, Asian Americans, and European Americans, compared to those without HCC.^167^ In the future, a deeper analysis of epigenetic factors, with respect to ethnic disparities, could be useful in providing an improved understanding the progression novel biomarkers, treatment options, and outcomes in HCC patients.

Overall, there is a significant variation in the underlying etiology, cancer stage at diagnosis, prognosis and mortality between different ethnic groups. Understanding these variations and implementing preventative and control strategies should improve patient outcomes and decrease these disparities.

## 4 VARIATIONS IN HCC TREATMENT AND INTERVENTION

It has been estimated that HCC causes 90% of all primary liver cancer diagnoses.^7^ Although HCC occurs throughout the world, it has an uneven distribution.^42^ Sub‐Saharan Africa and East Asia account for the highest number of age‐related HCC cases.^168^ In China, liver cancer accounts for approximately 50% of all cancer‐related deaths.^169^

Insufficient treatment resources and a lack of timely diagnosis can be linked to the HBV epidemic in Sub‐Saharan Africa.^170^ In these regions, less than 1% of HBV patients are diagnosed, although appropriate diagnostic methods are available.^171^ In Sub‐Saharan Africa, only 15% of patients that receive an early diagnosis of HBV go to hospitals and pharmacies for the required treatment.^171^ Tenofovir is one of the most commonly used drugs to treat HBV.^171^ Tenofovir, at a dosage of 80–300 mg/day (depending on certain patient factors), is one of the primary treatments for HBV.^172^ A study in Australia reported that tenofovir decreased the plasma levels of HBV by 71%, 80%, and 89%, after 12, 24, and 36 months of treatment, respectively.^173^ However, despite the efficacy of tenofovir against HBV, according to WHO, Sub‐Saharan Africa has  the second highest HBV burden in the world at 81 million people. This is compared to the Western Pacific region, which has the highest HBV burden at 116 million. Tenofovir is not utilized on a sufficient scale in these regions,^174^ which can be attributed to the high cost of tenofovir in the private sector, as well as the need for continual, lifelong treatments.^175^ The high rate of HBV transmission from mothers to children that occurs in nations, such as Africa, can be prevented using tenofovir, telbivudine, or lamivudine, during the last third trimester.^176^, ^177^, ^178^ A study in 1509 patients from Taiwan (6–26 years old) indicated that HBV vaccination in infants significantly decreased their risk of developing HCC.^140^ However, in 2017, only 19% of African nations administered HBV vaccines at birth.^179^ Thus, African nations with the highest levels of HBV infection should utilize the protective effects from vaccinations to prevent HBV, ultimately decreasing the incidence of HCC.

The comorbidity of HCC and HIV has been shown to greatly increase mortality, compared to having either disease alone.^180^ A meta‐analysis by Stabinski et al.^172^ indicated that HIV‐HBV co‐infection in sub‐Saharan African individuals ranged from 0% to 24.8%, with a median co‐infection rate of 7.8%. Furthermore, an increase in liver‐related mortality was reported by Thio et al.^173^ in HIV‐HBV co‐infected individuals with lower nadir CD4+ T cell counts of 100 cells/μL, compared to 250 cells/μL.^181^ The traditional therapeutic management of HCC may not be maximally efficacious because the response of CD4+ T cells in HBV infection is significantly decreased due to an HIV‐induced decrease in CD4+ T cell number.^182^ Furthermore, certain drugs used to treat HIV can produce hepatotoxicity, thereby increasing the risk of severe liver damage.^183^ A novel drug combination that may be useful for the treatment of HCV and HIV coinfection is elbasvir (an inhibitor of NS5A^184^), and grazoprevir (an inhibitor of HCV NS3/4A protease^185^), which together inhibit HCV replication. A clinical study of 218 patients indicated that treatment with a foxed dose tablet containing 100 mg a day of grazoprevir and 50 mg a day of elbasvir for 12 weeks produced a sustained virology response (SVR) of 96% without producing severe adverse effects.^186^ Similarly, the treatment of cirrhotic patients coinfected with HCV and HIV with pibrentasvir (an inhibitor of HCV NS5A^187^) and glecaprevir (an inhibitor of HCV NS3/4A protease^188^), produced an SVR of 98%.^189^ In a study of 335 patients, the combination of 90 mg of ledipasvir (an NS5A inhibitor^190^) and 400 mg of sofosbuvir (an inhibitor of HCV NS5B^191^), administered once a day for 12 weeks in patients with HCV genotype 1 or 4 produced an SVR of 96%.^192^

Globally, NAFLD occurs in approximately 25% of the population, with a range of 13% in Africa to 42% in Southeast Asia.^193^ A multicenter study of 1336 patients from 6 different countries (between 2005 and 2015) reported that 9% of the cases of HCC were secondary to NAFLD.^194^ It is predicted that by 2030, 50% of the population in the USA will be diagnosed as being obese.^195^ The prevalence of NAFLD is 1%–2% in the United States, whereas its prevalence is 7.6%–34.2% in the pediatric population worldwide.^196^, ^197^ It has been reported that up to 20% of patients diagnosed with NAFLD‐HCC undergo liver resection.^198^ NAFLD is common in South America and the Middle East, but it is rare in Africa.^199^ Diabetes, NASH, age, steatosis, and obesity are positively correlated with a diagnosis of NAFLD‐HCC, which increases the probability of liver failure after liver resection surgery.^200^ A recent study of 68 950 liver transplant patients in Europe reported a 1.2%–8.4% increase in the proportion of NASH patients receiving liver transplants from 2002 to 2016.^201^ When comparing geographical regions in the United States, the liver transplant ratio was 11.0% and 5%, in Caucasian Americans and African–Americans, respectively, in the southern states, compared to 7.2% and 3.8%, respectively, in non‐southern states.^16^ Mathur et al.^27^ reported the average survival rate in early‐stage HCC patients that were treated using invasive therapy for 5 years was 17.9%, based on all ethnic groups, with the rates for patients of African, Caucasian, Hispanic and of other ethnicities at 12.2%, 18.2%, 15.2% and 17.1%, respectively. Overall, 32.8% of patients received invasive therapy. African–American patients had a 12% higher mortality rate compared to Caucasian American patients, whereas Hispanic Americans had a similar mortality rate, and Asian Americans had a 16% lower mortality rate. This data indicates that the same treatment regimen produced different morality rates, depending on the ethnicity of the patient.^27^

Globally, despite the significant variations in the risk factors for HCC, the treatment regimens for HCC are virtually identical. Liver transplantation (LT) is an intervention that is used to treat certain patients with HCC. Approximately 15%–50% of patients with HCC undergo LT.^202^ The use of LT in early‐stage HCC patients, where the size of the tumor is 3 cm or less and there is an absence of invasion or metastasis, produces a 75% survival rate 4 years after transplantation, with a recurrence rate of 10%–15%.^203^ Clinical data indicates that LT is used less commonly in patients of African ethnicity, compared to Caucasians.^16^ One of the major issues regarding the use of LT is the scarcity of organ donors.^204^ Another approach that can be used in HCC patients is local ablation.^205^ Local ablative treatments are a less intrusive form of surgery that can be used in patients diagnosed with early stage disease.^206^ Furthermore, it can be used in management of various disease involving methods such as percutaneous ethanol injection, radiofrequency, and laser ablation.^207^ This approach has been shown to be associated with a lower risk of adverse effects and mortality, compared to surgical resection or transplantation.^208^ Patients diagnosed with unresectable, intermediate‐stage HCC can be treated using trans‐arterial chemoembolization (TACE).^209^ This treatment modality involves the administration of embolic‐inducing compounds within the blood vessels that ultimately reduce or abolish the tumor blood vessels.^210^ Furthermore, TACE prevents the loss of chemotherapeutic drugs from the tumors, thus concentrating the drug within the cancerous cells.^210^ A randomized control study reported that the median survival of patients is increased from 16% to 20% when TACE was used to treat various types of cancers, compared a to an oil‐in‐water emulsion.^211^ Another treatment, known as a drug‐eluting bead (DEB), has been reported to increase the efficacy of TACE therapy when used concurrently.^212^ DEB decreases the diffusion of drug to the peripheral circulation and increases the local drug concentration, which increases the anti‐tumor efficacy of TACE.^213^ The mortality rate from HCC is decreased by approximately 7% in patients who were treated using TACE, compare to non‐TACE treated patients.^214^

**FIGURE 3:**
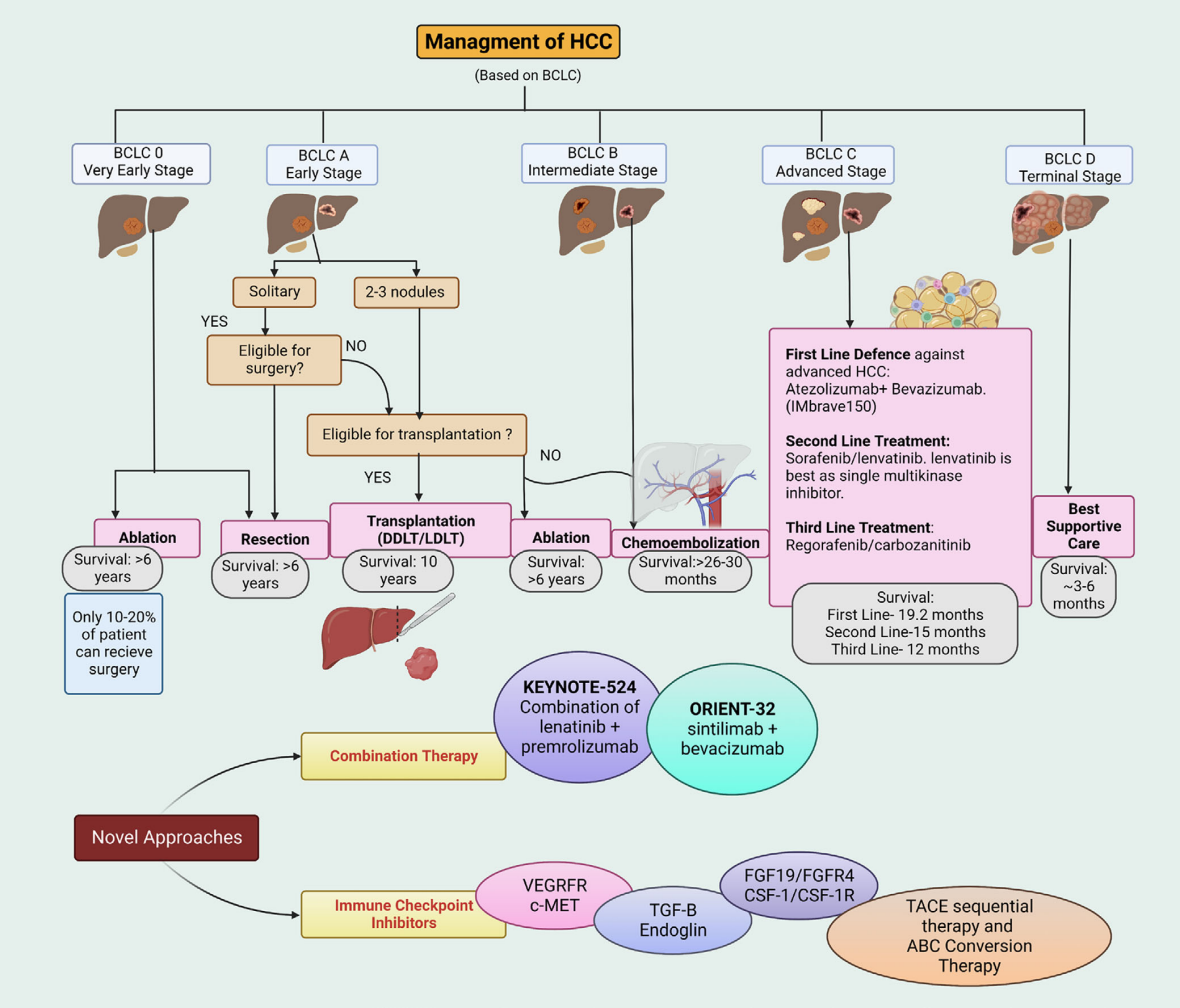
Management of HCC. There are many criteria that must be considered before initiating treatment in HCC patients. Barcelona Clinic Liver Cancer (BCLC) is a widely accepted classification, and it helps in determining which treatments to use. There are various factors, such as surgery eligibility, transplantation eligibility and the stage of HCC, among others.

Another approach for the treatment of HCC is the use of certain monoclonal antibodies (mABs). The U.S. FDA has approved the use of sorafenib, atezolizumab–bevacizumab, and lenvatinib as first‐line treatments for patients diagnosed with advanced stage HCC.^215^ Lenvatinib, an inhibitor of VEGF, FGF, and PDGF receptors,^216^ was reported to be efficacious in certain HCC patients.^217^ A Phase III, multicenter clinical trial was conducted in the Asian‐pacific, European, and North American regions, that compared the efficacy of lenvatinib to sorafenib at various oral doses in patients with untreated HCC. The dose of levantinib ranged from 8 to 12 mg/day which was given to 478 patients and 400 mg of sorafenib was given twice a day to 476 patients for a total of 28 days. The study concluded that lenvatinib was noninferior to sorafenib in the overall survival of untreated HCC patients. The most common adverse effects produced by lenvatinib were hypertension, diarrhea, decreased appetite, and weight loss, compared to erythrodysaesthesia in patients treated with sorafenib.^218^ In another phase III clinical trial, the efficacy of a 2:1 dose ratio of atezolizumab and bevacizumab was evaluated in 336 patients.^186^ The overall survival was 67.2% for patients treated with atezolizumab‐bevacizumab, compared to 54.6% for patients treated with sorafenib. The results indicated that the combination of atezolizumab and bevacizumab produce a significantly greater overall survival compared to sorafenib (NCT03434379). Nivolumab is a fully human immunoglobulin G4anti‐PD‐1 monoclonal antibody that has been approved for the treatment of certain cancers^219^, ^220^, ^221^, ^222^, ^223^, ^224^, ^225^ In September 2017, nivolumab was approved by the US FDA as a second line treatment for liver cancer in patients that did not respond to sorafenib.^226^ A phase III clinical study with 743 patients evaluated the efficacy and safety of 240 mg of intravenously nivolumab every 2 weeks, compared to 400 mg of sorafenib, twice daily, until disease progression or unacceptable toxicity was detected.^220^ Overall, the results indicated that nivolumab had favorable clinical outcomes and a good safety profile for the treatment of HCC. Although it did not significantly improve survival when compared to sorafenib, it provided a potential treatment for patients that cannot be treated with tyrosine kinase inhibitors.^221^ The efficacy of mAbs for the treatment of HCC is dependent upon age, progression of the disease, comorbidities, geographical location, and genetic variation. Figure 3 summarizes the BCBL classification of liver malignancy and its associated treatment algorithm, including several novel approaches for the treatment of HCC.

## 5 LIMITATIONS THAT PRODUCE DISPARITIES IN CLINICAL OUTCOMES FOR HCC PATIENTS

Ethnic and gender differences impact the prevalence, mortality and treatment outcomes of HCC.^28^ However, the scientific and medical community must look beyond only reporting disparities and identify and target the specific determinants that impact the incidence and progression of HCC. The multidimensional nature of HCC requires clinical and social interventions to address the complexity of diagnosis and treatment modalities. Ethnic and geographical disparities for HCC are well‐established^227^ and the lack of routine surveillance, which results in delayed diagnosis, is one of the key factors that produces the disparities in HCC.^228^ The poor prognosis for socio‐economically vulnerable populations is the result of various contextual and individual variables.^229^ Contextual‐level measures, also known as Social Determinants of Health (SDoH), include neighborhoods, access to healthcare insurance, education and poverty, significantly affect the clinical outcomes of HCC.^230^ Patients with nonprivate insurance have been shown to have significantly worse outcomes, even when thoroughly evaluated, regardless of their HCC stage at presentation.^231^ A recent study indicated that lack of healthcare insurance was a major factor in decreasing the overall survival (OS) in many patients.^232^ Indeed, a median OS of 34 months, compared to a median OS of 9‐months, has been reported for privately insured and noninsured patients, respectively.^231^ Nevertheless, having healthcare insurance does not necessarily result in improved outcomes. Out‐of‐pocket expenses, such as clinic visits, may affect anyone with limited financial capacity.^233^ Individual level barriers, such as culture, language and logistic barriers, including transportation and caretaker expense, are factors that significantly affect clinical outcomes.^158^ However, variations in access to healthcare do not fully explain the disparities seen in HCC. For example, epigenetic modulators such as obesity, viral hepatitis, diabetes, NAFLD, and chronic stress due to poverty and discrimination, impact the molecular basis of cancer and its prognosis.^234^ Healthcare providers and systems must make dedicated effort to provide equal care to all patients to improve therapeutic outcomes and decrease the disparities that occurs in HCC patients. The multifactorial etiology behind the disparities that alter the incidence and progression of HCC among different ethnic and geographical groups requires significant and specific considerations when determining the optimal treatment regimen.

## 6 CONCLUSION AND FUTURE GUIDANCE

Ethnic and geographical differences play a significant role in both the incidence and mortality rate of HCC. The poor prognosis for these patients is often a result of a late diagnosis due to inadequate screening measures. It is widely accepted that there are several factors that influence the heterogeneity of HCC. For example, HCV, HBV, age, sex, alcohol consumption, obesity, and genetics contribute to the various outcomes of HCC. Furthermore, a variety of factors can contribute to the sporadic development of HCC. In some populations, these factors are present to different extents and magnitudes. Understanding the various etiologies behind the development and progression of HCC between different populations may help with a timelier diagnosis and improve overall outcomes. While this issue is being thoroughly investigated, additional research will be required. Unfortunately, there are no reliable early biomarkers for HCC and preventative and other screening measures have not been fully implemented in certain countries. Several treatment options are unavailable to patients due to financial constraints and their overall scarcity. Improvements in early detection, effective and affordable treatment plans, and preventative measures can decrease the disparities that are present in ethnic and geographic populations of HCC patients.

Ethnic differences can have a significant impact on HCC assessment and treatment. Some ethnic groups have higher rates of HCC due to factors such as viral hepatitis infections, alcohol consumption, and nonalcoholic fatty liver disease. In addition, access to health care and resources may differ, which can impact the type of treatment and follow‐up care. When assessing HCC patients, ethnic differences must be taken into consideration to ensure effective treatment. This can include factors such as cultural beliefs, patient attitudes toward health care, patient preferences, and disparities in access to care. The use of standardized outcome measures and assessment tools should be culturally sensitive and validated. The impact of ethnicity and other demographic factors on treatment response and outcomes can also be better understood by collecting data.

To address the ethnic differences in HCC assessment and treatment across regions several factors can be considered. The development of international clinical practice guidelines that take into account patient populations, health systems, and resources can lead to global consensus. Through the use of evidence‐based clinical practice guidelines and standardized outcome measures, HCC patients can be assessed and treated more consistently. International clinical trials and registry data can also be used to determine the most effective treatments and assessment methods. By standardizing the assessment method and approach, it will be possible to compare results and outcomes between countries, improving patient care and advancing understanding of HCC. By assuring that all patients receive appropriate treatment and follow‐up care, improving the availability and quality of health care services in underserved communities can help address disparities in access to care. Health care providers can improve patient outcomes by providing education and training about the latest developments in the assessment and treatment of HCC, including culturally sensitive approaches. The collection and sharing of patient demographics, treatment response, and outcomes can improve our understanding of the impact of geographical location on HCC treatment. International sharing of this data could facilitate the development of effective and equitable treatments. Finally, the promotion of collaborations between health care providers, researchers, and patient advocacy groups can ultimately advance the understanding of liver cancer worldwide.

## AUTHOR CONTRIBUTIONS

**Vivek Chavda:** Conceptualization (equal); data curation (equal); investigation (equal); validation (equal); visualization (equal); writing – original draft (equal). **Kelsee K. Zajac:** Formal analysis (equal); methodology (equal); resources (equal); writing – original draft (equal). **Jenna Lynn Gunn:** Visualization (supporting); writing – original draft (supporting). **Pankti Balar:** Formal analysis (supporting); visualization (supporting); writing – original draft (supporting). **Avinash Khadela:** Validation (supporting); writing – original draft (supporting). **Dixa Vaghela:** Software (supporting); visualization (supporting); writing – original draft (supporting). **Shruti Soni:** Formal analysis (supporting); investigation (supporting); methodology (supporting); validation (supporting). **Charles R. Ashby:** Formal analysis (equal); investigation (equal); project administration (equal); supervision (equal); validation (equal); visualization (equal); writing – review and editing (equal). **Amit K. Tiwari:** Conceptualization (equal); formal analysis (equal); funding acquisition (equal); supervision (equal); writing – original draft (equal); writing – review and editing (equal).

## CONFLICT OF INTEREST STATEMENT

The authors have stated explicitly that there are no conflicts of interest in connection with this article.

## ETHICS STATEMENT

The work submitted to Cancer Reports has been done in accordance with these guidelines and that is has been performed in an ethical and responsible way, with no research misconduct, which includes, but is not limited to data fabrication and falsification, plagiarism, image manipulation, unethical research, biased reporting, authorship abuse, redundant or duplicate publication, and undeclared conflicts of interest.

## Data Availability

Data sharing is not applicable to this article as no new data were created or analyzed in this study.
